# Expression of AIF and Caspase-3 in New Zealand rabbit with Cervical Spondylosis Myelopathy model

**DOI:** 10.1016/j.amsu.2021.102604

**Published:** 2021-07-28

**Authors:** Sabri Ibrahim, Abdurrahman Mousa, Wibi Riawan

**Affiliations:** a Department of Neurosurgery, Faculty of Medicine, University of Sumatera Utara, Medan, Indonesia; b Department of Biochemistry and Pathology, Faculty of Medicine, Universitas Brawijaya, Malang, Indonesia

**Keywords:** Cervical spondylosis myelopathy, AIF, Caspase-3, Spine

## Abstract

**Introduction:**

Cervical spondylosis myelopathy (CSM) is a clinical syndrome of motoric or sensoric, caused by degenerative process chronically causing narrowing of cervical canal and compressing the spinal cord. The narrowing of canalis spinalis causing chronic compression and disrupting vascular patency in spinal cord. This is worsened on repetitive trauma on flexion, extension and rotation. CSM has an incidence of 4.04 in 100.000 cases per year and the total patients undergoing treatment operative or nonoperatively in year increasing for 7 times. Apoptosis plays a critical role in important biological processes such as morphogenesis, tissue homeostasis, and immunity; furthermore, its aberrant activation or impairment may contribute to a number of diseases. The understanding of CSM pathophysiology from apoptotic pathway is an essential topic to discussed and the treatment of this case in future.

**Method:**

This study uses experimental study with Post test Only Control Group, using New Zealand rabbits. The rabbits given the compression on cervical as high as C5 to induce CSM. The tissue was taken from spinal cord on compression area and histopathology examination was done to calculate apoptotic factor expression such as AIF and caspase-3.

**Result:**

Chronic compression on spinal cord causing myelopathy clinically on animal study, resulted in weakness of all extremities. Based on this study, the expression of AIF and caspase-3 is increasing in compression group in day 14 to day 21.

**Conclusion:**

Chronic compression in spinal cord causing increase in AIF and caspase-3 in day 14 and day 21, and this may be caused by increasing of apoptotic expression on animal study.

## Highlights

• Cervical Spondylosis Myelopathy.
• AIF.
• Caspase-3.
• Spine.
• Animal Model.

## 1 Introduction

Cervical spondylosis myelopathy (CSM) is a clinical syndrome caused by decreasing function in spinal cord, affecting motoric and sensoric, caused by chronic degenerative resulting in narrowing of vertebral canal and compression of spinal cord. The patient might complaint about paresthesia, progressive loss in motor function and balance function, in advance cases there is loss of function in defecation and micturition. Degenerative changes occurred in intervertebral disc, formation of osteophyte, hypertrophy of ligamentum flavum and facet joint. Compression can be occurred in one level or multi level [[1], [2], [3]] (see Graphic 1, Graphic 2, Graphic 3, Graphic 4, Graphic 5, Graphic 6, Graphic 7, Graphic 8)

**Graphic 1:**
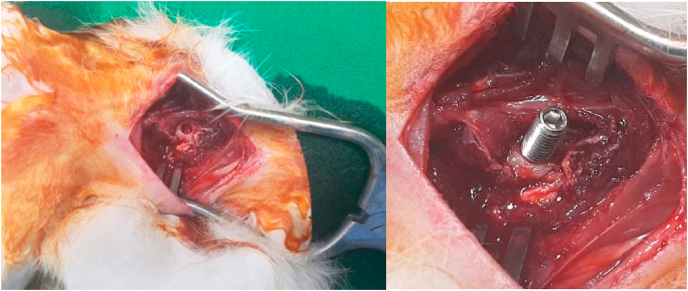
Procedure in screw placement in animal study.

**Graphic 2:**
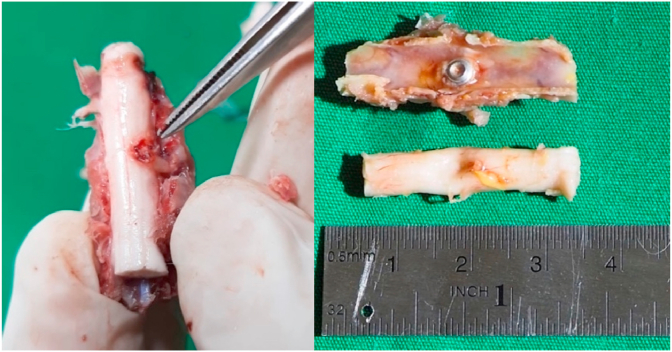
Screw in animal study.

**Graphic 3:**
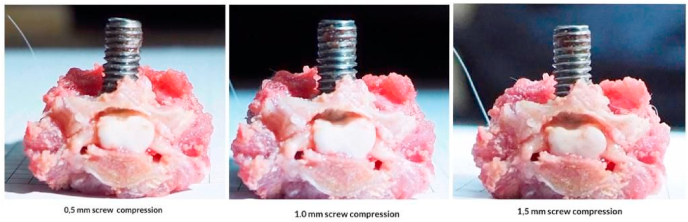
Screw compression placement.

**Graphic 4:**
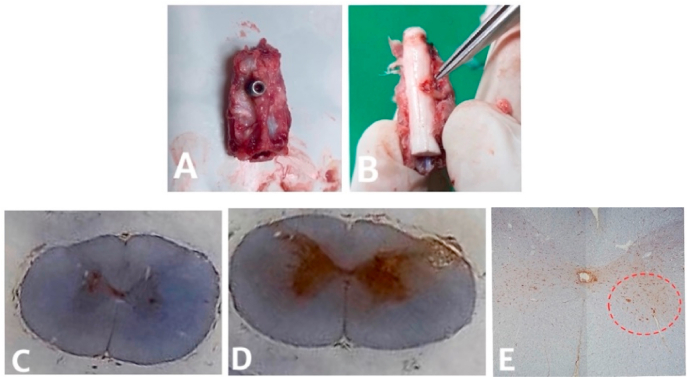
A. Vertebral cervical C4/5/6 B. spinal cord and area of compression C. Axial cut of spinal cord in C5 level of control group D. Spinal cord axial cut in compression level C5 E. Area of examination in anterior horn.

**Graphic 5:**
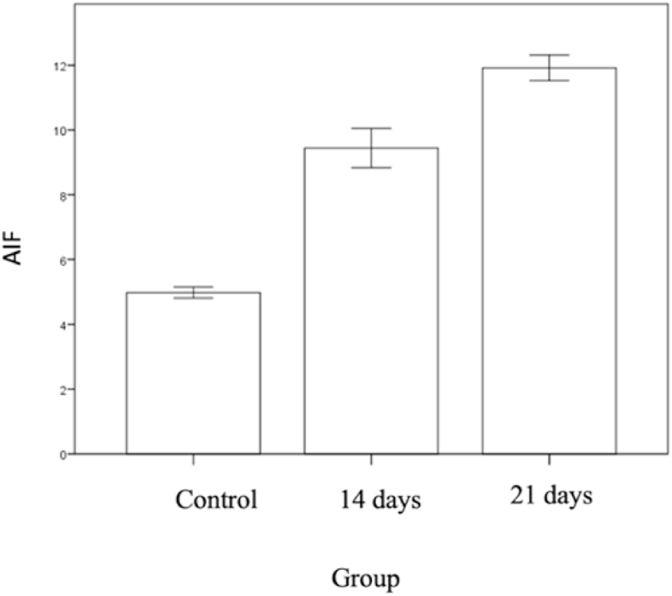
Left: total cell expressing AIF in control group and compression group 14 days and 21 days.

**Graphic 6:**
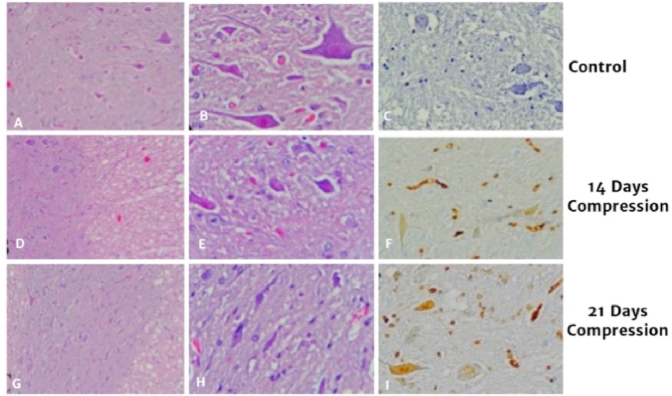
A,D,G before smear with HE was done, B,E,H after HE smear was done, C,F,I was done after immunohistochemistry examination.

**Graphic 7:**
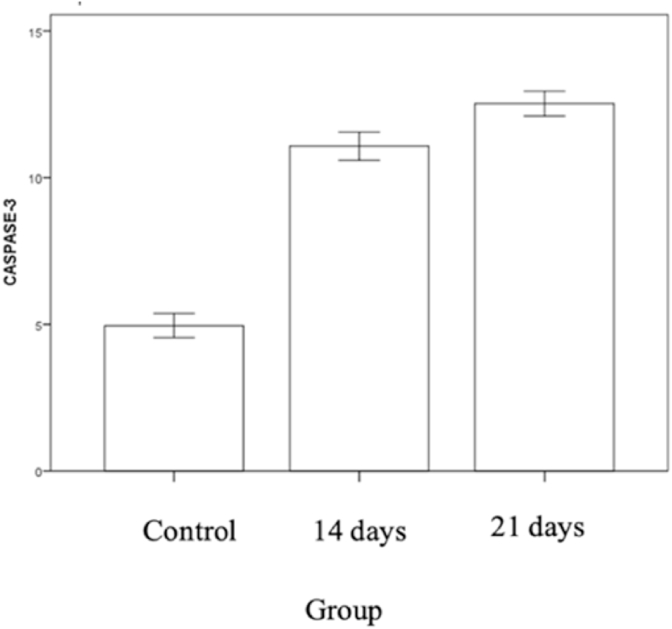
Total cell expressing caspase-3 in control group and compression group 14 days and 21 days.

**Graphic 8:**
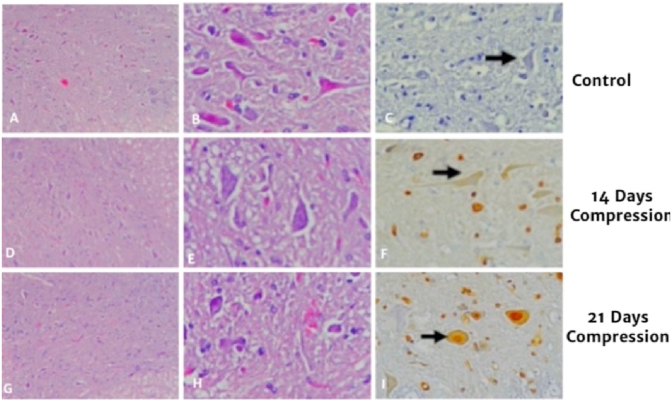
A,D,G before smear with HE was done, B,E,H after HE smear was done, C,F,I was done after immunohistochemistry examination.

Narrowing of spinal canal causing compression chronically and disrupting vascular patency in spinal cord, worsened by repetitive trauma on flexion, extension and rotation. OPLL is a condition with hyperostois of spine, in which posterior longitudinal ligament (PLL) calcify progressively, causing symptomatic spinal canal stenosis. Genetic factor, environment and biomechanic factor have been implicated on developing this case with high prevalence in Asia population. OPLL also has familial distribution, radiologically a quarter of family with OPLL is genetic [[1], [2], [3]].

Cervical spondylosis is the most common myelopathy in cervical caused by compression chronic in spinal cord in over 55 years old patient, related to CSM with incidence of 4.04 per 100.000 cases per year and total patient needing treatment operatively is increasing 7 times. Data shown that incidence and prevalence of CSM with compression of spinal cord in US is 4.10–6.05 in 100.000 cases. The retrospective study found that CSM is the most common diagnosis (23.6%) in 585 patients visiting UK Hospital with paraparesis and tetraparesis. Another study in UK reported that in 41 patients diagnosed with CSM, the mean age is 68.7 years. Another study in Japan from 100.000 population in Northeastern prefecture, 5 people diagnosed with CSM with age of 60–70 years old [4,5].

CSM can cause myriad sign and symptoms. Although there is no pathognomonic sign, the onset of disease is occult, with duration of 1 week until 26 years and almost half of patients when diagnosed have also one or more clinical sign. On study with 1.076 patients with CSM, there is instability in walking, spasticity, numbness in extremities and loss of control in hand motor. Another sign is pain in neck, radiating to shoulder or subscapular. One third patients with neck pain with CSM and cephalgia and two third with unilateral or bilateral shoulder pain [4,6,7].

The total patient complaining neck pain radiating to hand is also common, with sign of upper motor neuron such as hyper-reflex, clonus, babinski sign, defecation and micturition problem. Those clinical sign can occurred simultaneously with lower motor neuron sign such as hyporeflex and atrophy of upper extremity. Numbness or sensation impairment in upper extremity usually not specific, although sensation impairment in dermatome can occurred with radiculopathy. The changes in lower extremities also common and typically suggesting dorsal column involvement. Also, weakness in muscle also causing gait in CSM [[8], [9], [10]].

Diagnosis of CSM may involving cervical radiography, showing the formation of osteophyte, kyphosis, or even subluxation. Although that MRI spine is the most reliable diagnostic tool. MRI can also evaluating spinal cord, ligament, and intervertebral disc, and can also rule out pathologic such as spinal cord tumor or syrinx. Moreover, T2WI sequences shows that hyperintense on compression level in spinal cord showing the relation in severity of CSM and regarded as prognostic factor. Edema and inflammation also occurred. T1WI hypointense also showing more severe sign, resulting from ischemic, myelomalacia or gliosis related to worsening prognosis intraoperative. MRI can also has low sensitivity in detecting mild spinal cord damage, especially in patient with chronic disease [[7], [8], [9]].

Patient with no neurological deficit or no deteriorating clinical sign can also be treated conservatively and observed. Patient with moderate to severe disability on first examination is a strong candidate for decompression [7,8,11].

Apoptosis plays a critical role in important biological processes such as morphogenesis, tissue homeostasis, and immunity; furthermore, its aberrant activation or impairment may contribute to a number of diseases. Cells undergoing apoptosis usually exhibit a characteristic morphology, including apoptotic body formation, fragmentation of the cellular proteins, nuclear and cytoplasmic condensation, and chromosomal DNA cleavage [12,13].

Characteristics of apoptosis comprise both caspase-dependent and caspase-independent pathways. Apoptosis Inducing Factor (AIF) and Caspases are major mediators involved in several death pathways, such as extrinsic death receptor-mediated or intrinsic mitochondria-mediated signaling. Caspase activation affects a number of substrates that have important biological functions, leading to the loss of their functional roles [14,15].

Many author concluded that inflammation role in promoting and initiating the development of myelopathy in cervical spondylosis. Inflammation from mechanic compression in spinal cord or ischemic occurred. Many study concluded that the increase of IL-6 and TGF-β1 in CSF of CSM patient, they concluded that antiinflammation can be used as alternative treatment non operatively or prophylacyic strategy in degenerative cervical [16].

## 2 Method

### 2.1 Study design

This study is experimental with method of Post test Only Control Group Design. Ths study was done in standardized laboratory and also have complete equipment and experience on animal study, preparation and examination of histopathology smear and immunohistochemistry. This study was done in biochemistry laboratory in Brawijaya University and cutting the paraffin block was done in Pathology lab in Brawijaya University.

### 2.2 Sample

New Zealand rabbits with age of 12 weeks, weighted 2.6–3 kg and mean of 2.9 kg, male is used in this study. Animal study was given diet and water conventionally in lab. The temperature in cage is 16–20 °C with light dark cycle in 12 h. This study has been approved by ethical committee of faculty of medicine, Universitas Sumatera Utara Medan.

This study uses 15 rabbits and divided into 3 groups, the first group (n = 5) is the control group, incised in skin, dissected in paraspinal muscle, burr hole lamina and no laminar screw placement was done. Second group (n = 5) was given compression in spinal cord and laminar screw placement was done, and terminated in day 14. The third group (n = 5) was given spinal cord compression with laminar screw placement and terminated in day 21.

### 2.3 Procedure

Rabbit is on anesthesia using 50 mg/kgBW Ketamine hydrochloride (Pfizer) and 10 mg/kgBW Xylazine (Bayer)10. Rabbit in prone position, posterior area in cervical is shaved, disinfected with betadine 10%, closed with sterile cloth, incised in midline skin posterior cervical as high as C4–C6, small retractor, palpated in processus spinosus, dissecting the paraspinal muscle in C5, identify the lamina. A hole was made in lamina C5 in midline position using high speed drill diamond bur (3 mm diameter), until reaching lamina (thickness is 2 mm), the hole tapered 4 mm, and screw was placed with diameter of 4 mm and length of 10 mm, until all the lamina was through, on day 1 compression was given 0.5 mm (rotating screw 180°), on day 7 screw was rotated 180° (total compression is 1 mm), on day 14, screw was rotated again (compression total of 1,5 mm), after screw placement skin is suture, with position of 0,5 cm under the skin, palpable, and on day 2 and 3, the skin incision and screw can be manipulated easily, on repeated procedure, the sterile and anesthesia used is the same [17,18].

### 2.4 Tissue preparation

Spinal cord tissue in compression area fixated with buffer solution formalin 10%. After that, dehydration is done using alcohol (30%, 50%, 70%, 80%, 96% and absolute) 60 min respectively. Clearing is used with xylol twice with each given 60 min. Embedding is done with soft paraffin 60 min in 48 °C. Next, paraffin is isolated in a day until becoming hard block. The next day, holder was placed and cut into 4 μm with rotary microtome. Deparaffinisation with object glass from paraffin block in xylol twice in 5 min, and rehydrated with alcohol serial (absolute, 96%, 80%, 70%, 50% and 30%) in 5 min respectively then washed with H2O2 in 5 min, continued with washing the slide with PBS ph7,4 in 5 min. Next, using the Hematoxillin in 10 min. Using tap water in 10 min, and rinsed with dH2O2. Dehydration was done with serial alcohol 30% and 50% with 5 min respectively using Eosin in 3 min. Next, rinsed with alcohol 30%, washed with dH2O2 in 5 min and dried. Mounting was done with cover glass.

### 2.5 Tissue evaluation

Tissue evaluation with counting total cell with brown color in nuclear cell in anterior horn of compression area and counting of cell number was done separately (double blind). The examination and counting of cell number was done in each slide on first cortical spinal cord with magnification of 400x, each 20 times respectively.

## 3 Result

### 3.1 Motoric function

Clinical evaluation in animal study showed no sign of acute spinal cord injury. Sign that is shown was chronic myelopathy. Motoric level function in animal study given compression in spinal cord decreasing slowly until day 21. Motoric function in animal study before dissected in day 14 compression is 3, and on day 21 is 2. This result differ from animal study in control group with 4.

### 3.2 Macroscopic evaluation

On compression area, spinal cord showed flattened on anterior-posterior, referring to chronic compression. Histological evaluation with HE showed lesion typical of chronic myelopathy, ischemic changes and anterior horn alteration.

### 3.3 AIF (apoptosis inducing factor)

The mean value of cell expressing AIF in control group is 4.98 ± 0,90, compression group in 14 days is 9,44 ± 1,36 and compression group in 21 days is 11,92 ± 0,87. There is difference in total cell expressing AIF in control group and compression 14 day group (p = 0.001), there is also significant difference in cell expressing AIF in control and compression day 21 group (p = 0.001), there is a significant difference of cell expressing AIF in group compression 14 days with compression 21 days (p = 0.004).

### 3.4 Caspase-3 (cysteine-aspartic proteases)

The mean value of cell expressing caspase-3 in control group is 4.96 ± 0,92, compression group in 14 days is 11,08 ± 1,07 and compression group in 21 days is 12,52 ± 0,96. There is difference in total cell expressing caspase-3 in control group and compression 14 day group (p = 0.001), there is also significant difference in cell expressing AIF in control and compression 21 day group (p = 0.001), there is no significant difference of cell expressing AIF in group compression 14 days with compression 21 days (p = 0.009).

## 4 Discussion

Pathophysiology in spinal cord injury and cervical myelopathy has been known occurred parallelly. It was suggested that mechanical injury caused by static and dynamic comprising of compression inducing secondary injury on level of molecular. It was known that in injury of spinal cord, there is increase in expression of apoptotic factor such as AIF and caspase-3 [19].

In this study, expression of AIF increase in 14 day after compression and increasing gradually to day 21. The pathway of independent apoptosis was influenced by AIF, where pro-apoptotic translocated in nucleus and activated DNA degradation. Expression of AIF enough to create apoptosis.Aoptosis is one of many pathology in CSM^13,14,18.^

On this study, expression of caspase-3 increase until day 14 compression of spinal cord and also increasing gradually until day 21. Production of caspase inside cell inactive and have to undergo activation of proteolytic in apoptosis, activation of caspase-3 as executor done by initiator caspase resulting in cell death. Another study done also showed chronic compression of spinal cord in cervical resulting to apoptosis mediated by Fas Ligand on neuron cell and oligodendrogliocyte related to activation of caspase-3,-8 and -9 causing neurological deficit progressively [20,21]. On this study, there is increase in capase-3 expression.

The most significant biological changes in CSM is ischemic, BSCB disruption, chronic inflammation in neuron and apoptosis. On animal study, it has been done that the chronic compression on spinal cord so that the changes in pathologic and molecular mimicking CSM. This knowledge give potential and hope for the future for intervention, from intervention to definitive measure [17,18] Mechanical injury primarily caused by static and dynamic component such as compression, and retraction inducing secondary injury in molecular level^15.^

Apoptosis-inducing factor (AIF) has been identified as an apoptogenic mitochondrial intermembrane protein and has been demonstrated to play roles as a caspase-independent apoptotic factor. Caspases are crucial mediators of programmed cell death (apoptosis). Among them, caspase-3 is a frequently activated death protease, catalyzing the specific cleavage of many key cellular proteins [12,13]. The idea that AIF can induce caspase-independent death is based on several key pieces of evidence. Some reports, however, suggest that crosstalk between AIF and caspases is possible. At least in some cases, AIF has been reported as an essential apoptotic factor released from mitochondria in the Cyt-c-dependent caspase activation cascade [14,15].

## 5 Conclusion

Apoptosis-inducing factor (AIF) has been identified as an apoptogenic mitochondrial intermembrane protein and has been demonstrated to play roles as a caspase-independent apoptotic factor. Caspases are crucial mediators of programmed cell death (apoptosis). Chronic compression in spinal cord causing increase in apoptosis expression, this caused by the increase in AIF and caspase-3 on day 14 and day 21 after compression. Chronic compression of spinal cord causing myelopathy clinically on animal study, resulted in weakness of all extremities.

## Ethical approval

Approval has been given by ethical committee of Universitas Sumatera Utara.

## Sources of funding

None.

## Author contribution

Sabri Ibrahim: Author. Abdurrahman Mousa: Co- Author. Wibi RIawan: Co-Author.

## Registration of research studies

1. Name of the registry: None.

2. Unique Identifying number or registration ID: None.

3. Hyperlink to your specific registration (must be publicly accessible and will be checked): None.

## Guarantor

Sabri Ibrahim: Author.

Email: sabriibrahimnc@gmail.com.

## Consent

Not Applicable.

## Limitation of study

1. Longer observation is needed to ensure the marker is stable or decreasing on period of time.

2. Need the adjuvant of MRI spine and EMG to complete examination.

3. Another group needed to ensure the if the compression is treated, the expression of biomarker will decrease and clinically improving.

## Declaration of competing interest

There is no conflict of interest in this study.
